# P-Glycoprotein Induction Ameliorates Colistin Induced Nephrotoxicity in Cultured Human Proximal Tubular Cells

**DOI:** 10.1371/journal.pone.0136075

**Published:** 2015-08-19

**Authors:** Sun-hyo Lee, Jin-sun Kim, Kameswaran Ravichandran, Hyo-Wook Gil, Ho-yeon Song, Sae-yong Hong

**Affiliations:** 1 Department of Internal Medicine, Soonchunhyang University Cheonan Hospital, Cheonan, Republic of Korea; 2 Division of Renal Diseases and Hypertension, University of Colorado Denver, Aurora, Colorado, United States of America; 3 Department of Microbiology, College of Medicine, Soonchunhyang University, Cheonan, Republic of Korea; Indian Institute of Toxicology Reserach, INDIA

## Abstract

The pathogenesis of colistin induced nephrotoxicity is poorly understood. Currently there are no effective therapeutic or prophylactic agents available. This study was aimed to determine the mechanism of colistin induced nephrotoxicity and to determine whether P-glycoprotein (P-gp) induction could prevent colistin induced nephrotoxicity. Colistin induced cell toxicity in cultured human proximal tubular cells in both dose and time dependent manner. Colistin provoked ROS in a dose dependent manner as measured by DCF-DA. To investigate apoptosis, caspase 3/7 activity was determined. Caspase 3/7 activity was increased dose dependently (25, 50, 100 μg/ml) at 6 h. Autophagosome formation was assessed by measuring LC3- II/LC3-I ratio. The ratio of LC3-II to LC3- I was increased at 2 h (25 μg/ml). Suppression of autophagosome formation increased colistin induced nephrotoxicity. The expression of P-gp and the cell toxicity was determined in colistin with or without dexamethasone (P-gp inducer) and verapamil (selective P-gp inhibitor). Colistin itself suppressed the expression of P-gp. P-gp expression and activity decreased colistin induced nephrotoxicity with dexamethasone treatment. In addition induced P-gp transporter was shown to improve the efflux effect on colistin treated HK2 cell line, which was demonstrated by calcein-AM fluorescence accumulation assay. The increased activity could be blocked by N-acetylcysteine. In conclusion, colistin induces nephrotoxicity by suppressing P-gp. Induction of P-gp could ameliorate colistin induced nephrotoxicity by decreasing apoptosis.

## Introduction

Colistin (polymyxin E) is an important constituent of the polymyxin class of cationic polypeptide antibiotics. Its major components are colistin A (polymyxin E1) and colistin B (polymyxin E2) [1]. Colistin is administered to humans as colistin methane sulfonate (CMS), an inactive prodrug that requires conversion to colistin for antibacterial activity [2]. The use of intravenous CMS has been associated with nephrotoxicity [3,4]. It is now recognized that the incidence of nephrotoxicity with colistin is not as high as previously thought. However, there is no doubt that the administration of CMS has the potential to cause nephrotoxicity [5,6].

The nephrotoxicity mechanism has not been fully established, although increased cell membrane permeability, cell swelling and cell lysis associated with an increased influx of cations, anions, water and eventually apoptosis have been implicated [7]. Recent studies in rats suggest that oxidative stress and endoplasmic reticulum pathway plays a role in colistin induced nephrotoxicity [8–10]. Eadon et al. showed cell cycle arrest in a model of colistin induced nephrotoxicity [11]. Colistin can be transported into tubular cells, especially proximal tubular cell, by organic anion transporters [12,13]. But the excretion mechanism of colistin is yet to be revealed.

P-glycoprotein (P-gp), a membrane efflux pump confers a multidrug resistant phenotype to cancer cells by actively extruding a variety of structurally unrelated cytotoxic chemicals outside the cell [14]. The inhibition of P-gp can increase cell toxicity because P-gp is also involved in the secretion of substrates [15,16]. However, the role of P-gp in colistin induced nephrotoxicity has not been studied in detail. Therefore, the purpose of our study was to reveal the mechanism of colistin induced nephrotoxicity. We investigated that impact of P-gp induction on colistin induced nephrotoxicity in cultured human proximal tubular cells.

## Materials and Methods

### Chemicals and reagents

For *in vitro* study, colistin sulfate was purchased from Sigma Aldrich (St. Louis, MO, USA). All cell culture media and supplements were from Gibco (Invitrogen, Camarillo, CA, USA). Reagents for reverse transcription and those for real-time PCR reactions were from Toyobo (Osaka, Japan). Anti-LC3-I/II mouse monoclonal antibodies and a rabbit monoclonal antibody that detects endogenous levels of total β-actin protein were purchased from Cell Signaling Technology (Beverly, MA, USA). Secondary goat anti-rabbit IgG was obtained from Thermo Fisher Scientific (Rockford, USA). The assay kit for caspase-3/7 activity was purchased from Promega (Mannheim, Germany)

### Cell cultures

The immortalized proximal tubule epithelial cell line from normal adult human kidney (HK-2) was purchased from the American Type Cell Collection. Cells were grown in a humidified atmosphere at 37°C with 5% CO_2_ in a medium made of a 1:1 (vol/vol) mixture of Ham’s F-12 and Dulbecco’s modified Eagle’s medium supplemented with 10% Fetal bovine serum (Hyclone), 100 U/ml penicillin, and 10 mg/ml streptomycin (Hyclone).

### Cell viability

Cell viability was assessed using the 3-(4,5-dimethylthiazol-2-yl)-2,5-diphenyltetrazolium bromide (MTT) assay as described previously [17]. Briefly, sub-cultured cells (1 × 10^4^ cells/mL) were exposed to various concentrations of the test compounds in 24-well plate and incubated for 72 h at 37°C in 5% CO_2_. Following the 72 h incubation, 5 mg/mL MTT solution (Sigma) was added to the wells and cells were incubated for a further 4 h. The supernatant was then removed and 1 mL of DMSO was added to each well. Immediately after purple formazan crystals had formed and dissolved, the solution was collected and pipetted into a 96-well plate. The optical density was measured at 590 nm using the optical density at 630 nm as reference (VICTOR X3; PerkinElmer, USA).

### Assessment of cellular oxidative stress

Production of intracellular reactive oxygen species was detected using the nonfluorescent cell-permeating compound, 2′-7′-dichlorofluorescein diacetate (DCF-DA). DCF-DA is hydrolysed by intracellular esterases and then oxidized by ROS to a fluorescent compound, 2′-7′-dichlorofluorescein (DCF). After treatment with colistin, HK-2 cells were treated with DCF-DA (10 μM) for 30 min at 37°C. Following DCF-DA exposure, cells were rinsed and then scraped into PBS with 0.2% Triton X-100. Fluorescence was measured with a plate reader (VICTOR X3) with excitation at 485 nm and emission at 535 nm.

### RNA expression analysis of HK2-cells after colistin exposure

The Stress and Toxicity RT^2^ Profiler PCR Arrays (SABiosciences) and RT^2^ Real-Time SyBR Green/ROX PCR Mix were purchased from Qiagen. PCR was performed on ABI Prism 7900 HT (Applied Biosystems) according to the manufacturer's instructions. Samples from colistin treated and control HK2 cells were compared. For data analysis, the ΔΔCt method was used with the aid of a Microsoft excel spreadsheet containing algorithms provided by the manufacturer. Fold-changes were then calculated and expressed as log-normalized ratios of values from colistin treated/control.

### Apoptosis & autophagy measurement: caspase-3/7 activity and immunoblot assay for LC3-II/LC3-I ratio

Caspase-3/7 activity was detected using a Caspase-Glo 3/7 assay system (Promega) after preincubating the HK-2 cells (2 × 10^5^/96-well plate), followed by treatment with colistin concentrations (25 μg/ml, 50 μg/ml). The background luminescence associated with the cell culture and assay reagent (blank reaction) was subtracted from the experimental values. The activity of caspase-3/7 was presented as the mean value of triplets for given cells. The intensity of the emitted fluorescence was determined at a wavelength of 521 nm with the use of luminometer (VICTOR X3).

Immunoblots: After stimulation with dexamethasone (Dexa), cells were washed once with phosphate-buffered saline and lysed with radioimmunoprecipitation assay (RIPA) lysis buffer (ROCKLAND, USA) and placed on ice for 30 min. Total cell extracts were centrifuged at 14,000 g (for 20 min at 4°C). Protein-containing supernatants were collected. Equal amounts of proteins (40 μg) were resolved by SDS-PAGE gel electrophoresis, transferred to a nitrocellulose membrane and immunoblotted with specific antibodies against LC3-I/II and β-actin. Protein expression levels were quantified by densitometry (ChemiDoc XPS+with Image Lab Software, Bio-Rad). Data were represented as the ratio of expression of the target protein to that of β-actin.

### Acridine Orange Staining

Formation of acidic vesicular organelles, a morphological characteristic of autophagy was quantitated by acridine orange staining [18]. Acridine orange (0.5 mg/ml) (Invitrogen) was added 15 min prior to collection of cells. After washing with PBS, slides were analyzed with a confocal laser scanning microscope (Zeiss 410, Carl Zeiss GmbH, Gottingen Germany).

### Evaluation of MDR 1, ZO-1, occludin mRNA expression

Cells (5 × 10^5^ cells/ml) were seeded in 6-well plate, incubated for 24 h and then treated with the desired concentrations of drugs. After treatment, cells were washed with ice-cold PBS and harvested by trypsinization. Total cellular RNA was extracted using the RNeasy Mini Kit (Qiagen, Valencia, CA). Total RNA was quantified using a ND-1000 Spectrophotometer at 260 nm. The purity of the RNA was assessed using the 260 nm/280 nm ratio, which was always within 1.8–2.0, indicating high purity. 500 ng of each RNA sample was reverse transcribed using a random primer (Maxime RT Premix Kit, Intron biotechnology, Korea) in a Veriti 96-Well Thermal Cycler (Applied Biosystems, Carlsbad, CA). PCR was carried out using MDR1 specific primers as described by Taipalensuu et al. [19] and an iQ SYBR Green Supermix (Bio-Rad) kit in CFX96 Real-Time PCR Detection System (Bio-Rad). Reactions were performed in triplicates using the following protocol: 95°C for 5 min followed by 40 cycles of 95°C for 10 s, 42°C for 10 s, 72°C for 20 s. The primer pair sequences are as follow as: MDR1 F: CAGACAGCAGGAAATGAAGTTGAA, R: TGAAGACATTTCCAAGGCATCA; ZO-1 F: 5'TGGTGTCCTACCTAATTCAACTCA3', R: 5'CGCCAGCTACAAATATTCCAACA3', Occludin F: 5'TCCAGAGTCTTCCTATAAATCCAC3', R: 5'ACCACCGCTGCTGTAACG3', GAPDH F: ATGCAGCCGCATCTTCTT, R: GCCCAATACGACCAAATC. Assay results were normalized to the endogenous control GAPDH.

### P-gp function analysis

Cells were seeded at a density of 1 × 10^5^ cells/well (100 μl of culture medium) in a black-walled clear bottom 96-well plate (Nunc, Rochester, NY) overnight at 37°C. On the following day, 100 μl of desirable drug concentrations were added and incubated for predetermined time intervals. After incubation, tissue culture media were removed. Calcein AM stock solution was diluted in PBS to a concentration of 0.25 μM and 100 μl of this solution was used for each assay. The plate was then incubated at 37°C for 15–20 min. Intracellular accumulation of fluorescent calcein produced by calcein-AM hydrolysis through cellular esterase was measured using a Victor X3 multilabel reader (Perkin Elmer) at an excitation and emission wavelength of 485 nm and 535 nm, respectively. Both fluorescent and phase-contrast images were acquired using a Zeiss Axiovert-25 Microscope (Carl Zeiss GmbH, Gottingen Germany).

### Flow cytometry- propidium iodide (PI) and annexin V

Colistin-induced apoptotic and necrotic death of HK-2 cells was determined by annexin V and PI staining followed by flow cytometry. For staining of cells, the Dead Cell Apoptosis Kit with Alexa Fluor 488 annexin V and PI was used following the protocol provided by the manufacturer. Briefly, after treatments (colistin with or without Dexa, verapamil(VER), rapamycin(Rapa) or 3MA both floating and attached cells were pooled, washed and re-suspended in the Annexin V binding buffer. Alexa Fluor 488 annexin V and PI were added to suspended cells, and the reaction was incubated in the dark for 10 minutes. Flow cytometric analysis was performed by analyzing 10,000 gated cells utilizing the core service of Chungnam University. Untreated cells were taken as negative control. All experiments were performed at least 3 times.

### Statistical analysis

Results were expressed as means ± SEM (standard error of mean) from duplicate or triplicate samples of three independent experiments. Statistical significance was analyzed using one way ANOVA. Statistical significance was considered when *p* value was less than 0.05.

## Results

### Effect of colistin on cell viability and tight junction protein in HK2 cell line

**Fig 1:**
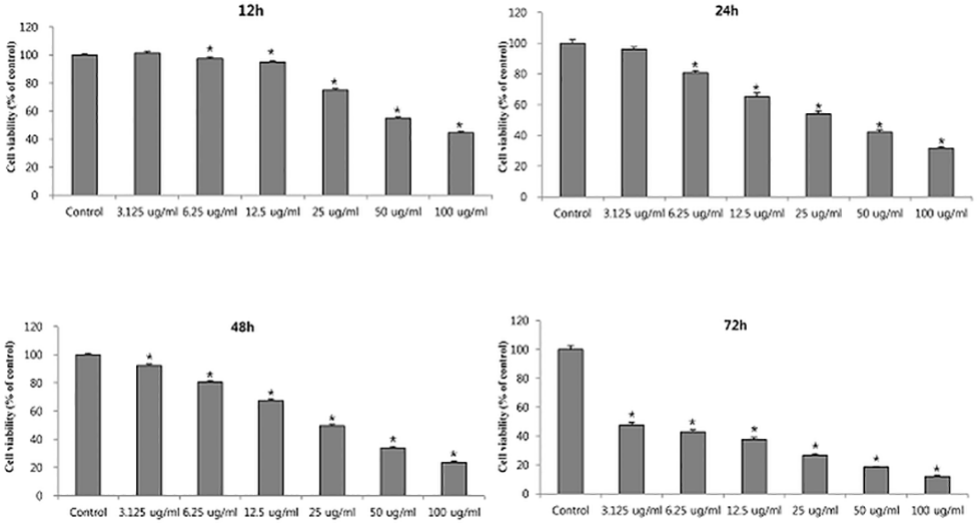
The effect of Colistin on HK-2 cell viability. The cell toxicity of colistin was found to be dose dependent. The data represented the mean ± SEM from 8 replicates in each group. *P < 0.01 at each time point compared to control.

**Fig 2:**
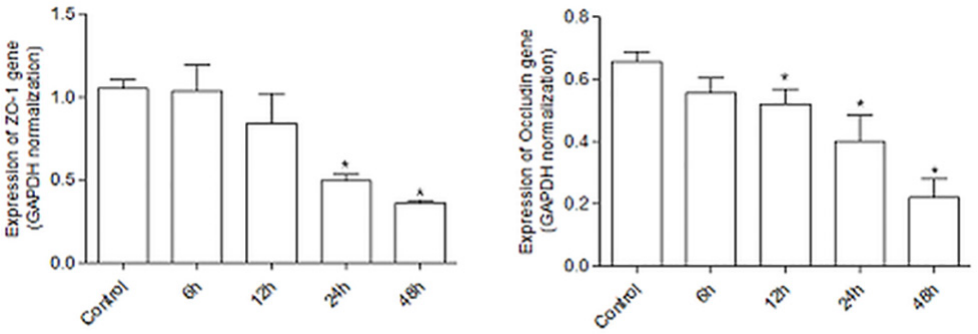
The effect of Colistin on tight junction mRNA expression in HK-2 cells. Colistin suppressed ZO-1 and occludin mRNA expression. The data represented the mean ± SEM from 3 replicates in each group. *P < 0.01 at each time points compared to control.

To determine the effect of colistin on cell proliferation of HK-2 cells, MTT assay was performed. As shown in Fig 1, colistin inhibited the cell proliferation of HK-2 in a dose dependent manner at each time point (12, 24, 48, and 72 h). Also we investigated the effect of colistin on tight junction mRNA expression. ZO-1 and occludin mRNA expression were suppressed after colistin (25 μg/ml) exposure (Fig 2).

### Colistin induced intracelluar oxidative stress

**Fig 3:**
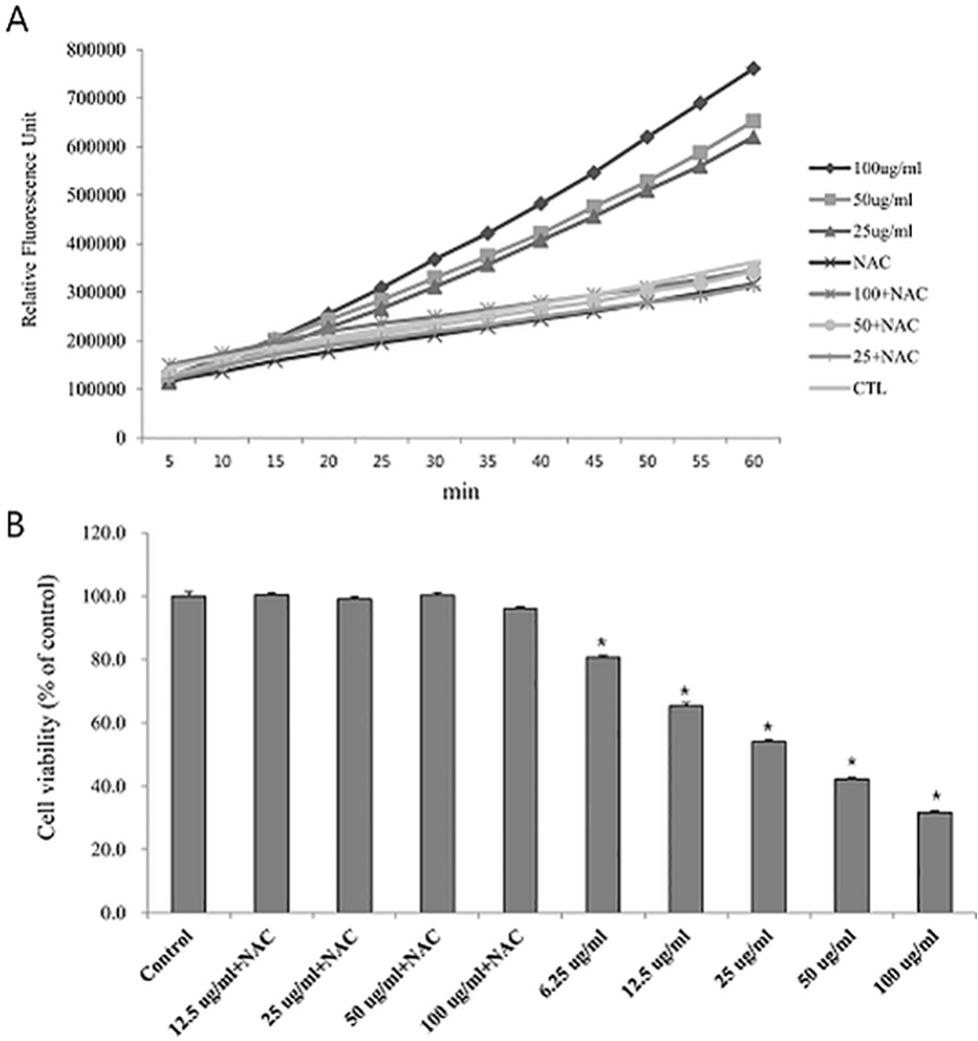
Colistin induced ROS in HK2-cell. (A) Time-dependent oxidation of DCF-DA in HK-2 cells by model oxidant compounds either in presence or absence of N-acetylcysteine (500 μM) & colistin. It was evident that colistin induced ROS production in HK-2 cells within 1 h. (B) N-acetylcysteine attenuated 24h colistin induced cell toxicity. The data represented the mean ± SEM from 8 replicates in each group. *P < 0.01 at each time point compared to control.

As shown in Fig 3A, colistin increased cellular oxidative stress in a concentration-dependent manner. Addition of antioxidants, N-acetylcysteine(NAC), suppressed free radical production. To evaluate ROS induced cytotoxicity, we investigated whether NAC reduced the cytotoxicity induced by colistin using MTT assay (Fig 3B). Our results revealed that NAC reduced colistin induced cell toxicity, suggesting that ROS mediated cytotoxic mechanism involved colistin induced cell toxicity.

### Role of P-gp in colistin induced nephrotoxicity

**Fig 4:**
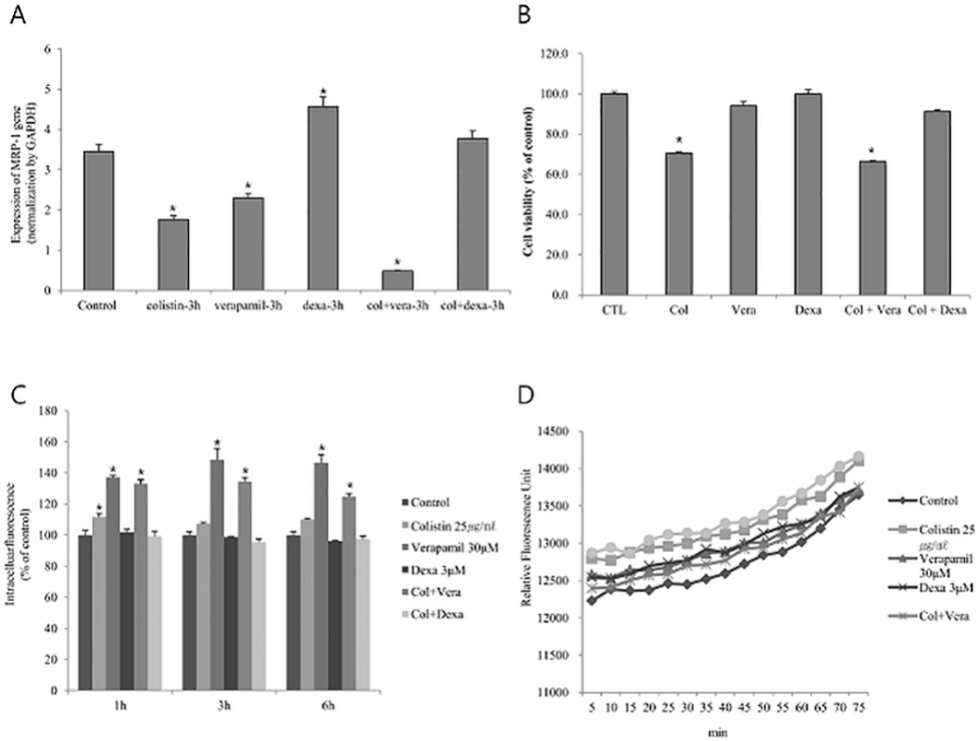
P-glycoprotein gene expression and effect of P-gp function on cell viability in colistin induced HK2 cells. (A) Expression of MDR1 mRNA in HK2 cells exposed to colistin 25 μg/ml, VER, Dexa, and colistin with VER or Dexa. 30 μM VER and 3 μM Dexa was used as control. Colistin 3h suppressed MDR 1 mRNA expression. Colistin with VER more suppressed MDR1 mRNA expression. Dexa attenuated colistin induced suppression of MDR1 mRNA expression. The bars presented means ± SEM of three independent experiments.*P < 0.05 vs. control cells at each time point.; (B) The cell survival percentage treated with colistin 25 μg/ml, VER 30 μM, Dexa 3 μM, and colistin with VER or Dexa. Dexa attenuated colistin induced cell toxicity; (C) Functional experiment of P-gp on Colistin induced HK-2 cells. In order to measure the activity of P-gp, the calcemic-AM assay was performed. A low efflux of the P-gp substrate in the calcein-AM assay was indicated by an increase in fluorescence. Colistin with VER increase calcein-AM assay by suppression of P-gp function; (D) ROS production was correlated with P-gp function.

P-gp (ABCB1, MDR1), a member of ATP-binding cassette (ABC) transporter family, is encoded by the human MDR1 gene. P-gp functions as a drug efflux pump under various conditions [20]. We hypothesized that the expression of P-gp could reduce the colistin induced cytotoxicity due to efflux of colistin. To test this hypothesis, we examined the expression of P-gp in HK-2 cell line (Fig 4A). Quantitative real-time PCR analysis demonstrated that colistin down-regulated MDR1 expression in HK2 cell line. However, MDR1 expression was not induced by verapamil (VER) (30 μM), a selective P-gp inhibitor (Fig 4A). VER aggregated colistin but down regulated MDR1 expression. MDR1 expression was induced by Dexa, a P-gp inducer. Dexa recovered colistin induced down regulation. In addition, Dexa reduced colsitin induced cell toxicity, while VER (30 μM) increased the toxicity of colistin (Fig 4B). We further investigated the functional role of the efflux pump P-gp in colistin treated HK-2 cells using calcein-AM accumulation and retention experiments. Cells were treated with colistin or VER and colistin. The accumulation of fluorescence calcein-AM was measured in the treated cells as a function of time (Fig 4C). VER with colistin resulted in the accumulation of intracellular fluorescence. VER, an inhibitor of P-gp, always increased the level of fluorescence, indicating a decreased level of functional P-gp. Dexa reduced the retention of fluorescence induced by colistin. These results suggested that P-gp function might involve the cell toxicity of colistin. In addition, the induction of P-gp reduced the production of ROS induced by colistin, whereas the inhibition of P-gp increased the production of ROS (Fig 4D).

### Relationship between autophagy and apoptosis in colistin induced nephrotoxicity

**Fig 5:**
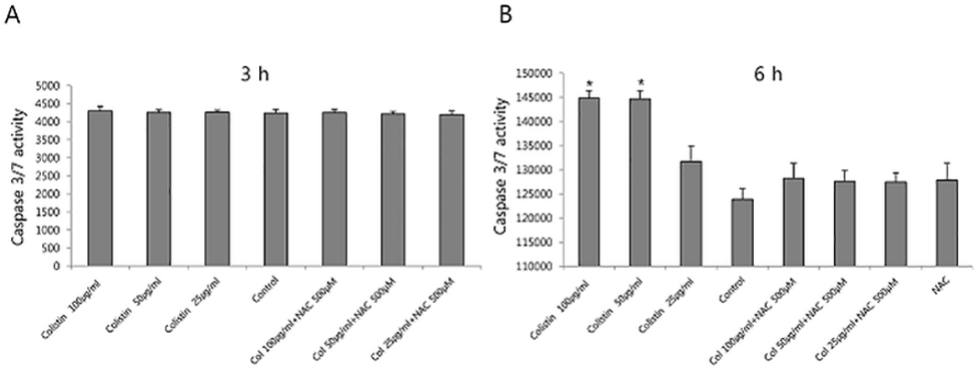
Effect of colistin on caspase-3/7 activity. Caspase-3/7 activity was analyzed using a Promega Caspase Glo 3/7 kit (described in the Materials and Methods). Caspase-3/7 activity was not increased at 3 h at each colistin concentration. After 6h, colistin (more than 50 μg/ml) increased caspase-3/7 activity. NAC (500 μM) antioxidant attenuated colistin-induced caspase-3/7 activity. The data represented the mean ± SEM (n = 6 for each group). *P < 0.05 vs. control cells at each time point.

**Fig 6:**
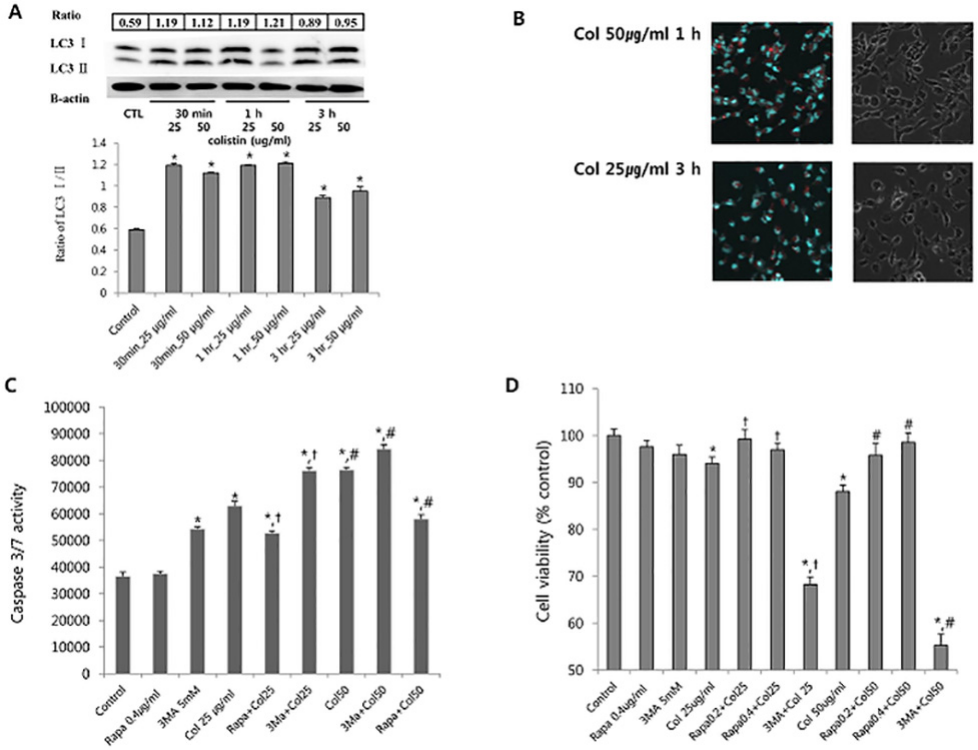
(A) Cell treated with colistin at each dose and each time. Immunoblot analysis of LC3 II. Samples containing 40 μg protein were loaded onto 15% SDS-PAGE gels. The blots were probed with the corresponding antibodies. The membrane was stripped and probed with β-actin. We calculated the ratio of LC3 II/I. The increased ratio suggested autophagosome formation; (B) Detection of colistin induced acidic autophagic vacuoles. Acidic vacuoles were detected (left panel, red vacuole). At 1 hr after colistin 50 μg/ml exposure, acid vacuole was observed. Right panel showing phase-contrast microscopy; (C) Rapa attenuated colistin induced caspase 3/7 activity, but 3MA accentuated colistin induced caspase 3/7 activity. Autophagy inhibited colistin induced caspase 3/7 activity. *P < 0.01 at each time point by one way ANOVA. *P < 0.05 vs. control, † P< 0.05 vs. colistin 25 μg/ml, # P<0.05 vs. colistin 50 μg/ml; (D) Pretreatment of Rapa, induced autophagy, prevented colistin induced toxicity. Co-treatment of 3 MA, which prevent autophagy, increased colistin toxicity. *P < 0.05 vs. control cell, † P< 0.05 vs. colistin 25 μg/ml, # P<0.05 vs. colistin 50 μg/ml.

**Fig 7:**
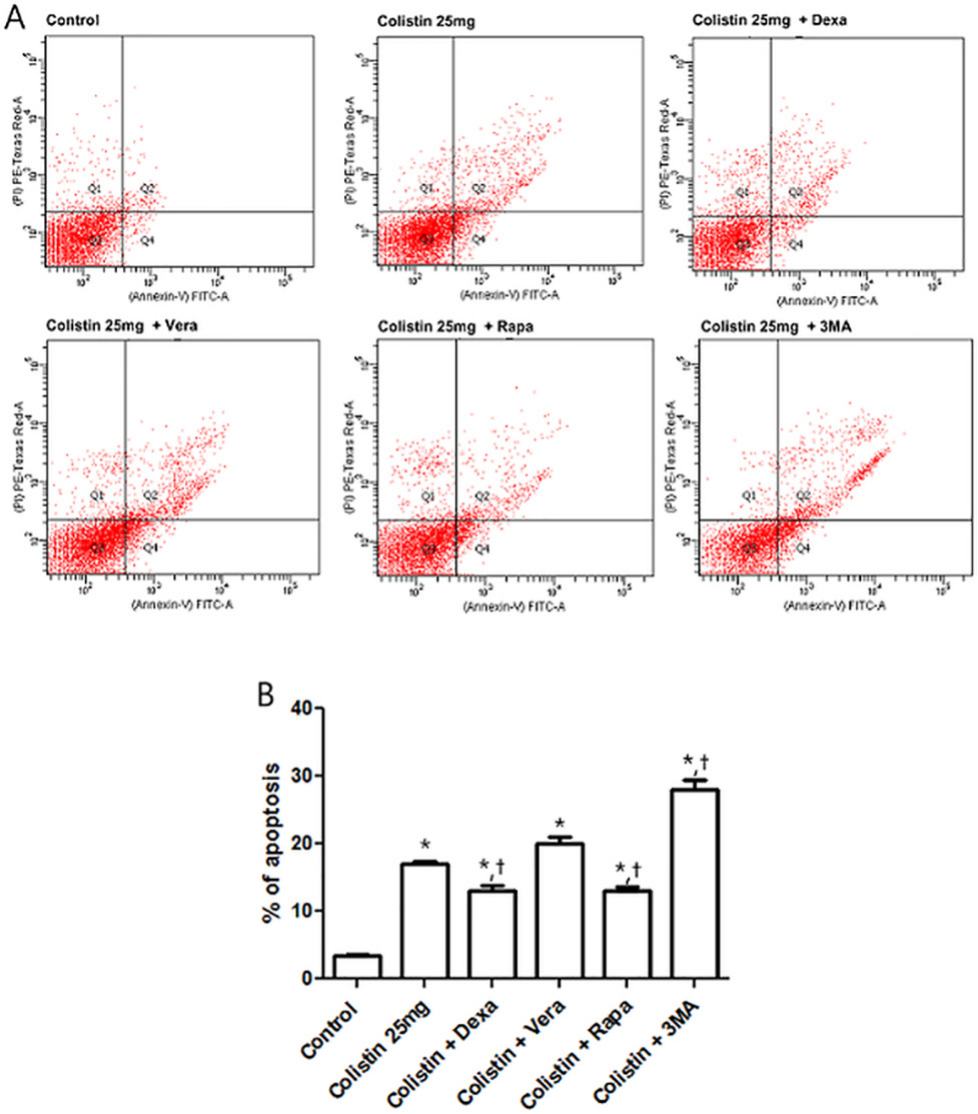
Effect of colistin on HK-2 cell apoptosis, determined by FACS analysis. (A) Cells were incubated with colistin for 24 h, after which they were harvested, the DNA was stained with propidium iodide, and the cells were analyzed using FACS. (B) Colistin increased the proportion of apoptotic cells in HK-2 cells. P-gp inducer and autophagy inducer attenuated colistin induced apoptosis. *P<0.05 vs. control cells. The data represent the mean ± SEM (n = 3 for each group).

We hypothesized that autophagy could play a protective role in colistin-induced nephrotoxicity. First, we investigated other oxidative stress related molecules that were affected by colistin using the Stress and Toxicity RT^2^ Profiler PCR Arrays. The full set of genes used in the PCR Arrays are listed in Table 1. Autophagy genes were elevated. These findings suggested the autophagy was associated with the mechanism of colistin induced nephrotoxicity. To evaluate apoptosis, we measured caspase 3/7 activity (Fig 5). Caspase 3/7 was not activated until 3 h following colistin exposure. At 6 h, caspase 3/7 was activated. The conversion of LC3-I to LC3-II is considered a reliable marker of autophagy. To determine the occurrence of autophagy during colistin nephrotoxicity, we examined LC3-II, a marker of autophagosome formation. LC3-II/LC3-I ratio was increased at 3 h of colistin treatment and then decreased toward basal levels (Fig 6A). Rapa at 0.2 and 0.5 mg/dl, the positive control also increased autophagy (data not shown). We also determined the effect of colistin on the formation of autophagolysosomes by fluorescence microscopy following staining with acridine orange. Cells treated with colistin displayed considerable red fluorescence because autophagolysosomes was acidic (Fig 6B). To test the effect of autophagy induction or inhibition on apoptosis, caspase 3/7 activity was measured after Rapa or 3MA addition to colistin treated HK-2 cells (Fig 6C). Autophagy induction with Rapa attenuate colisitn induced caspase 3/7 activity. Autophagy inhibition accentuated caspase 3/7 activity induced by colistin. Also, pretreatment of Rapa attenuated colistin toxicity, but blocking autophagy accelerated colistin toxicity (Fig 6D). To confirm these findings, the cells were incubated with colistin for 24 h, stained and subjected to FACS analysis to measure apoptosis and necrosis (Fig 7). Colistin increased the proportion of apoptotic cells. P-gp inducer and autophagy induction attenuated colistin induced apoptosis. The results suggest that autophagy is involved in the protection against colistin induced apoptosis.

**Table 1:** PCR Array RT^2^Profiler results showing genes that were significantly upregulated (The dose of colistin was 25 μg/ml).

GeneBank accession no.	Gene name	Symbol	Fold change	
			3h	6h
	*oxidative/metabolic stress*			
NM_003329	Thioredoxin	TXN	-1.05	-1.45
NM_001498	Glutamate-cysteine ligase, catalytic subunit	GCLC	1.27	1.36
NM_002061	Glutamate-cysteine ligase, modifier subunit	GCLM	-1.26	1.01
NM_000637	Glutathione reductase	GSR	1.64	1.44
NM_000852	Glutathione S-transferase pi 1	GSTP1	-1.33	1.09
NM_002133	Heme oxygenase (decycling) 1	HMOX1	1.61	1.95
NM_000903	NAD(P)H dehydrogenase, quinone 1	NQO1	-1.69	-1.03
NM_002574	Peroxiredoxin 1	PRDX1	-1.51	-1.10
NM_003330	Thioredoxin reductase 1	TXNRD1	**2.99**	**4.67**
NM_003900	Sequestosome 1	SQSTM1	1.25	1.40
NM_002032	Ferritin, heavy polypeptide 1	FTH1	1.67	1.63
	*Cell DEATH (Apoptosis signaling)*			
NM_003842	Tumor necrosis factor receptor superfamily, member 10b	TNFRSF10B(DR5)	1.42	1.75
NM_001065	Tumor necrosis factor receptor superfamily, member 1A	TNFRSF1A	-1.39	1.94
NM_021960	Myeloid cell leukemia sequence 1 (BCL2-related)	MCL1	**2.99**	**2.78**
NM_003844	Tumor necrosis factor receptor superfamily, member 10a	TNFRSF10A	-1.33	1.17
NM_033292	Caspase 1, apoptosis-related cysteine peptidase (interleukin 1, beta, convertase)	CASP1(ICE)	1.91	1.67
NM_000043	Fas (TNF receptor superfamily, member 6)	FAS	**2.20**	**4.21**
	*Cell DEATH (Autophagy signaling)*			
NM_004849	ATG5 autophagy related 5 homolog (S. cerevisiae)	ATG5	1.93	1.73
NM_006395	ATG7 autophagy related 7 homolog (S. cerevisiae)	ATG7	**7.06**	**8.08**
NM_004707	ATG12 autophagy related 12 homolog (S. cerevisiae)	ATG12	1.67	1.94
NM_003766	Beclin 1, autophagy related	BECN1	1.15	1.16
NM_000043	Fas (TNF receptor superfamily, member 6)	FAS	**2.20**	**4.21**
	*Cell DEATH (Necrosis signaling)*			
NM_017853	Thioredoxin-like 4B	TXNL4B	**9.06**	**11.03**
NM_002086	Growth factor receptor-bound protein 2	GRB2	1.52	1.38
NM_001618	Poly (ADP-ribose) polymerase 1	PARP1(ADPRT1)	1.21	1.57
NM_006505	Poliovirus receptor	PVR	**-2.07**	-1.51
NM_003804	Receptor (TNFRSF)-interacting serine-threonine kinase 1	RIPK1	1.15	1.17
NM_003844	Tumor necrosis factor receptor superfamily, member 10a	TNFRSF10A	-1.33	1.17
NM_003842	Tumor necrosis factor receptor superfamily, member 1A	TNFRSF1A	-1.39	1.94
NM_000043	Fas (TNF receptor superfamily, member 6)	FAS	**2.20**	**4.21**

## Discussion

Nephrotoxicity is the major adverse effect limiting the dose and duration of treatment with colistin [2,3,5]. Current studies have reported that renal toxicity due to colistin could be related to oxidative injury, which might be prevented by the use of some antioxidants [7–10]. But the exact mechanism of colistin toxicity is not well understood. Our toxicity data (Figs 1 and 2) suggested that colistin have time and dose dependent cell toxicity with the involvement of suppression of tight junction. Previous study suggested that colistin could enter the proximal cell via CAT [12] which could evoke ROS. Our study also showed that ROS was involved in colistin induced nephrotoxicity. Addition of antioxidant attenuated colistin-induced ROS production. The results suggest that ROS is one of the targets for treatment.

The immortalized cell line HK-2 maintains the normal phenotype of human renal tubule and expression of MDR1-P-gp. It is a valuable *in vitro* model to study P-gp modulation by xenobiotics and drugs [15,16]. By using this experiment system, we showed that P-gp transport activity was dose dependently inhibited by colistin. The induction of P-gp ameliorated the colistin induced nephrotoxicity. These findings suggest that P-gp might be an important transport protein to efflux colistin out of the cell. We used Dexa as a P-gp enhancer. However, *in vivo* Dexa is doubtful in enhancing P-gp in the kidney [21]. If kidney selective P-gp inducer would be investigated, that would improve clearance of colistin and reduce nephrotoxicity.

**Fig 8:**
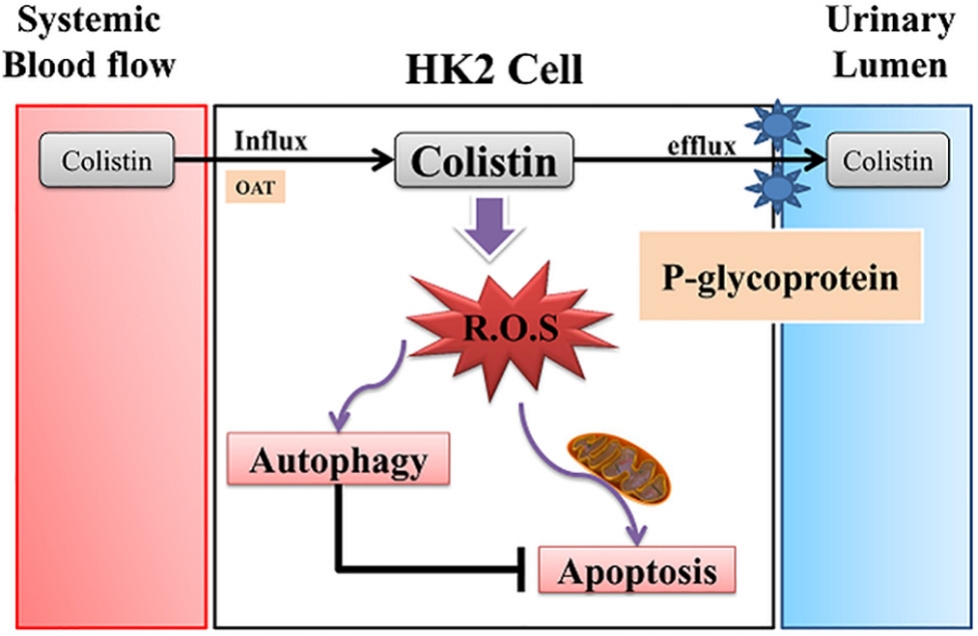
Schematic diagram of the proposed preventive mechanism of colistin induced toxicity. Colistin influx via organic cation transport evokes reactive oxygen species involved in apoptosis and autophagy. P-gp acts to efflux colistin from tubule cell. Enhancement of P-gp prevents colistin toxicity.

Autophagy is a physiologically regulated and evolutionary conserved process that plays a critical role in degradation of cytoplasmic proteins and other macromolecules within the lysosome in multicellular organisms [22–24]. Autophagy regulates cell death in both physiological as well as pathophysiological conditions [23,25]. Recently, it was reported that autophagy was activated in acute kidney injury induced by cyclosporine, cisplatin, and ischemic reperfusion [26–29]. This might be an important protective mechanism for renal tubular epithelial cell survival [30]. Our PCR array data suggested that colistin toxicity is related to autophagy, apoptosis and necrosis signaling. We speculated that the time could be important factor to influence death signal. We provide evidence that autophagy is an early response to colistin injury. Inhibition of autophagy by specific inhibitors increased caspase 3/7 activity and cell death, whereas induction of autophagy attenuated colistin induced caspase 3/7 activity and cell death. These findings suggest that the induction of autophagy plays a defense role against colistin. Our FACS data confirmed that P-gp and autophagy is involved with colistin induced nephrotoxicity. The mechanism of colistin induced nephrotoxicity is schematically proposed (Fig 8). In brief, strategies to prevent colistin induced nephrotoxicity include enhancement of efflux via P-gp induction, reduction of colistin induced ROS, and enhancement of autophagy.

In conclusion, colistin induced nephrotoxicity is involved by suppression of P-gp. Induction of P-gp could alleviate colistin induced nephrotoxicity by decreasing apoptosis. Further *in vivo* studies are needed to reveal the effect of these target treatments for colistin induced nephrotoxicity.

## References

[1] Li J, Nation RL, Turnidge JD, Milne RW, Coulthard K, Rayner CR, et al (2006) Colistin: the re-emerging antibiotic for multidrug-resistant Gram-negative bacterial infections. Lancet Infect Dis 6: 589–601. 16931410 10.1016/S1473-3099(06)70580-1

[2] Bergen PJ, Li J, Rayner CR, Nation RL (2006) Colistin methanesulfonate is an inactive prodrug of colistin against Pseudomonas aeruginosa. Antimicrob Agents Chemother 50: 1953–1958. 16723551 10.1128/AAC.00035-06 PMC1479097

[3] Ko H, Jeon M, Choo E, Lee E, Kim T, Jun JB, et al (2011) Early acute kidney injury is a risk factor that predicts mortality in patients treated with colistin. Nephron Clin Pract 117: c284–288. 10.1159/000320746 20847571

[4] Ghlissi Z, Hakim A, Mnif H, Ayadi FM, Zeghal K, Rebai T, et al (2013) Evaluation of colistin nephrotoxicity administered at different doses in the rat model. Ren Fail 35: 1130–1135. 10.3109/0886022X.2013.815091 23879363

[5] Falagas ME, Kasiakou SK (2006) Toxicity of polymyxins: a systematic review of the evidence from old and recent studies. Crit Care 10: R27 16507149 10.1186/cc3995 PMC1550802

[6] Phe K, Lee Y, McDaneld PM, Prasad N, Yin T, Figueroa DA, et al (2014) Comparison of nephrotoxicity rates associated with colistimethate versus polymyxin B therapy: in vitro assessment and a multicenter cohort study. Antimicrob Agents Chemother 58: 2740–2746. 10.1128/AAC.02476-13 24566187 PMC3993221

[7] Yousef JM, Chen G, Hill PA, Nation RL, Li J (2012) Ascorbic acid protects against the nephrotoxicity and apoptosis caused by colistin and affects its pharmacokinetics. J Antimicrob Chemother 67: 452–459. 10.1093/jac/dkr483 22127588 PMC3254197

[8] Ozyilmaz E, Ebinc FA, Derici U, Gulbahar O, Goktas G, Elmas C, et al (2011) Could nephrotoxicity due to colistin be ameliorated with the use of N-acetylcysteine? Intensive Care Med 37: 141–146. 10.1007/s00134-010-2038-7 20845026

[9] Yousef JM, Chen G, Hill PA, Nation RL, Li J (2011) Melatonin attenuates colistin-induced nephrotoxicity in rats. Antimicrob Agents Chemother 55: 4044–4049. 10.1128/AAC.00328-11 21709095 PMC3165279

[10] Dai C, Li J, Tang S, Li J, Xiao X (2014) Colistin-induced nephrotoxicity in mice involves the mitochondrial, death receptor, and endoplasmic reticulum pathways. Antimicrob Agents Chemother 58: 4075–4085. 10.1128/AAC.00070-14 24798292 PMC4068542

[11] Eadon MT, Hack BK, Alexander JJ, Xu C, Dolan ME, Cunningham PN (2013) Cell cycle arrest in a model of colistin nephrotoxicity. Physiol Genomics 45: 877–888. 10.1152/physiolgenomics.00076.2013 23922129 PMC3798781

[12] Ma Z, Wang J, Nation RL, Li J, Turnidge JD, Coulthard K, et al (2009) Renal disposition of colistin in the isolated perfused rat kidney. Antimicrob Agents Chemother 53: 2857–2864. 10.1128/AAC.00030-09 19380593 PMC2704651

[13] Yun B, Azad MA, Wang J, Nation RL, Thompson PE, Roberts KD, et al (2015) Imaging the distribution of polymyxins in the kidney. J Antimicrob Chemother 70: 827–829. 10.1093/jac/dku441 25377569 PMC4319485

[14] Gottesman MM, Pastan I (1993) Biochemistry of multidrug resistance mediated by the multidrug transporter. Annu Rev Biochem 62: 385–427. 8102521 10.1146/annurev.bi.62.070193.002125

[15] Romiti N, Tramonti G, Chieli E (2002) Influence of different chemicals on MDR-1 P-glycoprotein expression and activity in the HK-2 proximal tubular cell line. Toxicol Appl Pharmacol 183: 83–91. 12387747

[16] del Moral RG, Olmo A, Aguilar M, O'Valle F (1998) P glycoprotein: a new mechanism to control drug-induced nephrotoxicity. Exp Nephrol 6: 89–97. 9567214 10.1159/000020510

[17] Kim YH, Kwak KA, Gil HW, Song HY, Hong SY (2013) Indoxyl sulfate promotes apoptosis in cultured osteoblast cells. BMC Pharmacol Toxicol 14: 60 10.1186/2050-6511-14-60 24289746 PMC4222141

[18] Paglin S, Hollister T, Delohery T, Hackett N, McMahill M, Sphicas E, et al (2001) A novel response of cancer cells to radiation involves autophagy and formation of acidic vesicles. Cancer Res 61: 439–444. 11212227

[19] Taipalensuu J, Tornblom H, Lindberg G, Einarsson C, Sjoqvist F, Melhus H, et al (2001) Correlation of gene expression of ten drug efflux proteins of the ATP-binding cassette transporter family in normal human jejunum and in human intestinal epithelial Caco-2 cell monolayers. J Pharmacol Exp Ther 299: 164–170. 11561076

[20] Leslie EM, Deeley RG, Cole SP (2005) Multidrug resistance proteins: role of P-glycoprotein, MRP1, MRP2, and BCRP (ABCG2) in tissue defense. Toxicol Appl Pharmacol 204: 216–237. 15845415 10.1016/j.taap.2004.10.012

[21] Demeule M, Jodoin J, Beaulieu E, Brossard M, Beliveau R (1999) Dexamethasone modulation of multidrug transporters in normal tissues. FEBS Lett 442: 208–214. 9929003 10.1016/s0014-5793(98)01663-9

[22] Gozuacik D, Kimchi A (2007) Autophagy and cell death. Curr Top Dev Biol 78: 217–245. 17338918 10.1016/S0070-2153(06)78006-1

[23] Kundu M, Thompson CB (2008) Autophagy: basic principles and relevance to disease. Annu Rev Pathol 3: 427–455. 18039129 10.1146/annurev.pathmechdis.2.010506.091842

[24] Maiuri MC, Zalckvar E, Kimchi A, Kroemer G (2007) Self-eating and self-killing: crosstalk between autophagy and apoptosis. Nat Rev Mol Cell Biol 8: 741–752. 17717517 10.1038/nrm2239

[25] Levine B, Yuan J (2005) Autophagy in cell death: an innocent convict? J Clin Invest 115: 2679–2688. 16200202 10.1172/JCI26390 PMC1236698

[26] Zhang X, Howell GM, Guo L, Collage RD, Loughran PA, Zuckerbraun BS, et al (2014) CaMKIV-Dependent Preservation of mTOR Expression Is Required for Autophagy during Lipopolysaccharide-Induced Inflammation and Acute Kidney Injury. J Immunol 193: 2405–2415. 10.4049/jimmunol.1302798 25070845 PMC4215705

[27] Livingston MJ, Dong Z (2014) Autophagy in acute kidney injury. Semin Nephrol 34: 17–26. 10.1016/j.semnephrol.2013.11.004 24485026 PMC3984003

[28] Wang LT, Chen BL, Wu CT, Huang KH, Chiang CK, Hwa Liu S(2013) Protective role of AMP-activated protein kinase-evoked autophagy on an in vitro model of ischemia/reperfusion-induced renal tubular cell injury. PLoS One 8: e79814 10.1371/journal.pone.0079814 24223196 PMC3819246

[29] Yang C, Kaushal V, Shah SV, Kaushal GP (2008) Autophagy is associated with apoptosis in cisplatin injury to renal tubular epithelial cells. Am J Physiol Renal Physiol 294: F777–787. 10.1152/ajprenal.00590.2007 18256309

[30] Li L, Zepeda-Orozco D, Black R, Lin F (2010) Autophagy is a component of epithelial cell fate in obstructive uropathy. Am J Pathol 176: 1767–1778. 10.2353/ajpath.2010.090345 20150430 PMC2843468

